# Retraction Note: Inhibited MicroRNA-301 Restrains Angiogenesis and Cell Growth in Esophageal Squamous Cell Carcinoma by Elevating PTEN

**DOI:** 10.1186/s11671-023-03829-1

**Published:** 2023-03-20

**Authors:** Bin Wang, Peiyan Hua, Ruimin Wang, Jindong Li, Guangxin Zhang, Chengyan Jin, Yan Zhang

**Affiliations:** 1 Department of Thoracic Surgery, The Second Hospital of Jilin University, No. 218 Ziqiang Street, Changchun, 130041 Jilin China; 2 Department of Operating Room, The Second Hospital of Jilin University, Changchun, 130041 Jilin China

**Retraction note: Nanoscale Res Lett (2021) 16:3** 10.1186/s11671-020-03452-4

The Editors in Chief have retracted this article. After publication, concerns were raised about the appearance of the Western blots along with the following issues:

Unlikely appearance of the data presented in the flow cytometry histograms in Fig. 5A/E, in particular the unusually-looking peaks;

In Table 1 "Primer sequence", the putative PTEN primers appear to align to NF kappa B instead.

The authors provided quantification data for the Western blots but were unable to provide uncropped gels and blots. The Editors, therefore, have lost confidence in the integrity of the article's findings. The authors did not explicitly state whether they agree to this retraction.

